# Lineage Diversification and Evolutionary Dynamics of the *Hemagglutinin–Neuraminidase* Gene in Mumps Virus Genotype G

**DOI:** 10.3390/microorganisms14071597

**Published:** 2026-07-22

**Authors:** Fuminori Mizukoshi, Miu Takada, Ryusuke Kimura, Wei Liu, Yasuyoshi Hatayama, Mayuko Nishi, Yuka Sato-Fujimoto, Akira Kimura, Fumihiro Kato, Kei Miyakawa, Hirokazu Kimura, Akihide Ryo

**Affiliations:** 1 Department of Bioinformatics and Integrative Omics, National Institute of Infectious Diseases, Japan Institute for Health Security, Musashimurayama-shi 208-0011, Tokyo, Japan; mizukoshi.f@jihs.go.jp (F.M.); liu.w@jihs.go.jp (W.L.); hatayama.y@jihs.go.jp (Y.H.); nishi.m@jihs.go.jp (M.N.); 2 Department of Health Science, Graduate School of Health Sciences, Gunma Paz University, Takasaki-shi 370-0006, Gunma, Japan; t.miumiu501@icloud.com (M.T.); a-kimura@paz.ac.jp (A.K.); 3 Department of Health Science, Gunma Prefectural Institute of Public Health and Environmental Sciences, Maebashi-shi 371-0052, Gunma, Japan; kimura-r56@pref.gunma.lg.jp; 4 Faculty of Healthcare, Tokyo Healthcare University, Setagaya-ku 141-8648, Tokyo, Japan; y-fujimoto@thcu.ac.jp; 5 Center for Quality Management Systems, National Institute of Infectious Diseases, Japan Institute for Health Security, Musashimurayama-shi 208-0011, Tokyo, Japan; kato.f@jihs.go.jp; 6 Influenza Research Center, National Institute of Infectious Diseases, Japan Institute for Health Security, Musashimurayama-shi 208-0011, Tokyo, Japan; miyakawa.k@jihs.go.jp; 7 Institute for Vaccine Research and Development, Hokkaido University, Sapporo 001-0021, Hokkaido, Japan; 8 Advanced Medical Science Research Center, Gunma Paz University, Takasaki-shi 370-0006, Gunma, Japan

**Keywords:** Mumps virus, genotype G, *hemagglutinin–neuraminidase* gene, molecular evolution, phylodynamics, purifying selection, B-cell epitope prediction

## Abstract

Mumps virus (MuV) genotype G is widely represented among circulating strains, but the evolutionary patterns of the *hemagglutinin–neuraminidase* (*HN*) gene remain incompletely understood. In this study, we analyzed publicly available full-length genotype G *HN* sequences using phylogenetic, phylodynamic, codon-based selection, and structure-guided epitope prediction approaches. The genotype G *HN* sequences were categorized into Clade 1, an operationally defined Diverse group, and Clade 2. Clade 1, which was composed mainly of Japanese strains, showed a relatively structured pattern over time. In contrast, Clade 2 showed more recent diversification and an overall increase in relative genetic diversity, although this phylodynamic pattern was sensitive to sampling structure. The Diverse group was phylogenetically heterogeneous, and its Bayesian skyline estimates were not used for biological interpretation because repeated analyses showed unstable posterior behavior. Root-to-tip regression supported temporal structure in the complete dataset, with the strongest signal in Clade 2. Bayesian molecular dating estimated the time to the most recent common ancestor of the sampled genotype G *HN* sequences at approximately 1932, and the mean evolutionary rate was 4.925 × 10^−4^ substitutions/site/year. Although the HN protein is a major surface antigen and a target of neutralizing antibodies, codon-based analyses showed no robust evidence of positive selection using multiple methods. Instead, many codon sites were inferred to be under purifying selection, suggesting that genotype G HN evolution is largely constrained by the need to maintain protein function. Predicted B-cell epitope regions were broadly similar among representative genotype G strains. Overall, these findings indicate that genotype G *HN* lineages have followed distinct evolutionary patterns, while the *HN* gene remains mainly shaped by purifying selection. These findings may help improve our understanding of MuV genotype G *HN* gene evolution and support future molecular surveillance.

## 1. Introduction

Mumps virus (MuV) is an enveloped virus with a non-segmented, negative-sense single-stranded RNA genome and is classified within the family *Paramyxoviridae* and the genus *Orthorubulavirus* [1,2]. MuV is primarily transmitted through respiratory droplets and direct contact, initially replicating in the upper respiratory mucosa before disseminating systemically via viremia [2]. Infection typically manifests as salivary gland inflammation, particularly parotitis, and may be accompanied by complications such as orchitis, oophoritis, pancreatitis, and central nervous system involvement, especially in postpubertal individuals [2].

The MuV genome is approximately 15 kb in length and encodes nine proteins, among which the fusion protein (F) and the hemagglutinin–neuraminidase (HN) protein are expressed on the viral surface and play critical roles in viral entry and spread [2,3]. While the F protein mediates membrane fusion and enables viral penetration and cell-to-cell transmission, viral attachment to host cells and subsequent release are primarily governed by the HN protein through its sialic acid-binding and neuraminidase activities [3,4,5]. Notably, the HN protein constitutes a major target of neutralizing antibodies and is therefore a key determinant of host immune recognition and protective immunity [3,6,7].

From a molecular epidemiological perspective, MuV genotypes have traditionally been classified based on sequence variation in the SH gene, which encodes the small hydrophobic protein and represents a highly variable genomic region commonly used for MuV genotype assignment [8,9]. Based on SH gene variation, MuV is divided into multiple genotypes (A–N, excluding E and M), with genotype G predominating among circulating strains worldwide, particularly among sequences available from recent surveillance studies [8,9]. Although MuV is considered to comprise a single serotype, genetic variation within surface or antigenically relevant proteins may contribute to differences in immune recognition among viral lineages [10,11,12,13].

In this context, the HN protein deserves particular attention from the standpoint of viral antigenicity and host immune recognition. Compared with other viral proteins, the HN protein is exposed on the viral surface and is involved in both receptor binding and neutralizing antibody recognition, making it particularly informative for evolutionary and immune-related analyses [4,6,7]. Unlike the *SH* gene, which is primarily used for genotyping and provides limited insight into antigenic properties, the *HN* gene encodes a surface protein directly involved in host immune recognition and may therefore provide additional information on immune-related viral evolution [8,9,12]. In this context, increasing attention should be directed toward the *HN* gene and HN protein, given their central roles in viral attachment, antigenicity, and immune interaction. Despite their biological and immunological importance, previous studies have largely focused on *SH* gene-based genotyping, *HN* gene fragment analyses, or whole-genome sequencing, with relatively few investigations comprehensively addressing the molecular evolution of the *HN* gene itself [8,9,14,15]. In particular, systematic analyses using globally distributed MuV strains across extended temporal and geographic scales to elucidate evolutionary dynamics, selective constraints, and predicted epitope features of the *HN* gene remain limited.

To address these gaps, the present study employed a comprehensive bioinformatics framework to investigate the molecular evolution of the MuV *HN* gene and HN protein using globally distributed viral sequences. Phylogenetic reconstruction, molecular dating, Bayesian skyline plot analysis, evolutionary rate estimation, codon-based selection pressure analysis, and three-dimensional structure-based epitope prediction were integrated to characterize the genetic diversity, temporal dynamics, selective constraints, and predicted epitope features of the *HN* gene and encoded HN protein. By providing a global and systematic assessment of *HN* gene evolution, this study aims to improve our understanding of MuV evolutionary dynamics and to support future surveillance strategies.

## 2. Materials and Methods

### 2.1. Compilation and Curation of Publicly Available MuV HN Gene Sequences

Publicly available MuV *HN* gene sequences were retrieved from NCBI Virus (https://www.ncbi.nlm.nih.gov/labs/virus/vssi/#/) on 2 July 2024. Sequences were collected using two search queries: “Mumps virus genotype G, taxid:1384672” and “Mumps orthorubulavirus, taxid:2560602”.

Only sequences containing the complete coding region of the *HN* gene were included in the dataset. Sequences with ambiguous nucleotides, incomplete *HN* coding regions, frameshifts, premature stop codons, or unclear sampling information were excluded. Briefly, 10,197 records were retrieved using the genotype G-specific query, from which 853 complete *HN* sequences were retained after filtering and curation. In parallel, 13,998 records were retrieved using the broader species-level query, and 942 *HN*/*SH*-containing genotype G-confirmed sequences were retained after filtering and SH-based genotype confirmation. Among these, 240 sequences were not included in the genotype G-specific dataset and were therefore added to the dataset. After merging the 853 genotype G-query-derived sequences and the 240 additional sequences from the broader species-level query, 1093 sequences were obtained. Following inspection of sequence quality, metadata, and usability, 1090 sequences were retained, and one additional sequence was excluded because of inconsistent genotype assignment in the *SH* phylogenetic analysis. Thus, 1089 eligible complete genotype G *HN* sequences were retained before deduplication. The complete sequence-selection workflow is summarized in Supplementary Table S8. To account for the different requirements of molecular dating and sequence-comparison analyses, two deduplicated datasets were generated from these 1089 eligible sequences. First, a year-stratified deduplicated dataset was prepared by removing sequences that were identical and collected in the same year while retaining identical sequences collected in different years. This dataset was used for maximum-likelihood phylogenetic analysis, clock-like signal assessment, Bayesian time-calibrated phylogeny, Bayesian skyline plot analysis, evolutionary rate estimation, and median-joining haplotype network reconstruction. Second, a unique sequence dataset was generated by collapsing 100% identical *HN* gene sequences irrespective of sampling year, retaining the earliest available strain as the representative sequence. This dataset was used for analyses focused on genetic divergence and codon-based selection analysis.

Detailed information on the sequences used in this study, including accession numbers, strain names, sampling years, geographic origins, clade assignments, and inclusion in each dataset, is provided in Supplementary Table S1.

### 2.2. Maximum-Likelihood Phylogenetic Analysis of HN Gene Sequences

The full-length *HN* coding sequences in the year-stratified deduplicated dataset were used for maximum-likelihood phylogenetic analysis after removing the terminal stop codon. Multiple sequence alignment was performed using MAFFT version 7.520 [16]. The resulting alignment was inspected to confirm that the coding frame was maintained and to identify apparent alignment errors. Maximum-likelihood phylogenetic inference was then performed using IQ-TREE version 2.4.0 [17]. The best-fitting nucleotide substitution model was selected with ModelFinder implemented in IQ-TREE [18]. Branch support was evaluated using 1000 ultrafast bootstrap replicates and 1000 Shimodaira-Hasegawa-like approximate likelihood ratio test (SH-aLRT) replicates [19]. The principal IQ-TREE command and options were as follows: iqtree2 -s <alignment.fas> -m MFP -B 1000 -alrt 1000 -T AUTO. The inferred tree was visualized using FigTree version 1.4. Major genetic groups were assigned primarily based on the maximum-likelihood tree topology and node support values, and the consistency of these assignments was subsequently evaluated using patristic distance distributions and the median-joining haplotype network. The major node separating Clade 1 from the remaining genotype G *HN* sequences was supported by SH-aLRT/ultrafast bootstrap values of 87.7/99, and the major node separating Clade 2 from the intervening heterogeneous sequences was supported by values of 98.9/97. Clade 1 and Clade 2 also showed relatively narrow within-group patristic distance distributions and distinct positions in the haplotype network. In contrast, the Diverse group was not defined as a single well-supported monophyletic lineage. Instead, the term “Diverse group” was used operationally to refer to phylogenetically heterogeneous genotype G *HN* sequences located outside Clade 1 and Clade 2. Therefore, group assignments in this study were used as descriptive categories for comparative analyses and were not interpreted uniformly as strict bootstrap-supported monophyletic clades. Clade2-1, Clade2-2, and Clade2-3 were treated as subgroups within Clade 2, mainly reflecting tree topology, haplotype-network structure, and temporal/geographic patterns.

### 2.3. Quantification of Patristic Distances Within and Between HN Gene Lineages

To quantify genetic divergence within and between the major *HN* gene lineages, patristic distances were calculated using a maximum-likelihood tree separately inferred from the unique sequence dataset, following the same phylogenetic procedure described above. Pairwise patristic distances were obtained from the Newick-formatted tree using the Patristic program v1.0 [20]. Distances were grouped as within-lineage comparisons for the Diverse group, Clade 1, and Clade 2, or as between-lineage comparisons among these groups. The resulting distance distributions were visualized as violin plots using Orange Data Mining version 3.35 [21].

### 2.4. Median-Joining Haplotype Network Analysis of HN Gene Sequences

To visualize haplotype-level relationships among MuV *HN* gene lineages, a median-joining haplotype network was constructed using the year-stratified deduplicated dataset. The network was generated in PopART version 1.7 using the median-joining algorithm [22]. Haplotypes were defined based on identical *HN* gene sequences, and the size of each node was scaled according to the number of sequences assigned to that haplotype. Sequences were color-coded by country or region of origin to show the geographic composition of each haplotype. The genotype A vaccine/reference strain Jeryl-Lynn was included for contextual comparison.

### 2.5. Evaluation of Temporal Signal in the HN Gene Dataset

Before conducting Bayesian time-calibrated phylogenetic analysis, the temporal signal of the *HN* gene dataset was evaluated by root-to-tip regression using TempEst version 1.5.3 [23]. The year-stratified deduplicated dataset was used for this analysis because this dataset retained identical *HN* sequences when they were collected in different years. Maximum-likelihood trees were inferred from the complete *HN* gene dataset and from the major phylogenetic groups, including the Diverse group, Clade 1, and Clade 2, using the same tree inference procedure described above.

Sampling years were assigned to the corresponding tips of each maximum-likelihood tree. The association between sampling year and root-to-tip genetic divergence was then examined for the complete dataset and for each major group. The strength of the temporal signal was assessed using the coefficient of determination (R^2^) obtained from the root-to-tip regression analysis. TempEst was operated through the graphical user interface; after importing the IQ-TREE maximum-likelihood trees and assigning sampling years as tip dates, root-to-tip regression was performed for each dataset, and the resulting R^2^ values were recorded.

### 2.6. Bayesian Molecular Dating and Time-Calibrated Phylogeny

Bayesian time-calibrated phylogenetic analysis was performed for the complete genotype G *HN* gene dataset using BEAST2 version 2.7.6 [24]. The year-stratified deduplicated dataset was used for this analysis, and the aligned full-length *HN* coding sequences, excluding the terminal stop codon, were used as input. Sampling years were assigned to the corresponding sequences as tip dates.

The nucleotide substitution model was selected using jModelTest2 version2.1.10 (v20160303) [25]. To determine an appropriate model configuration for Bayesian molecular dating, nested sampling was used to compare combinations of six molecular clock models, including strict clock, random local clock, optimized relaxed clock, relaxed clock exponential, relaxed clock lognormal, and fast relaxed clock lognormal, and two coalescent tree priors, namely constant population and exponential population models [26]. Nested-sampling model comparisons were performed to estimate log marginal likelihoods for each candidate combination of molecular clock model and coalescent tree prior. The model with the highest log marginal likelihood was selected for the final BEAST2 analysis. The selected model configuration is summarized in Supplementary Table S2, and the log marginal likelihood estimates from nested sampling are provided in Supplementary Table S10.

Based on the selected model configuration, Markov chain Monte Carlo analysis was run for 300 million steps, with parameters sampled every 1000 steps. Convergence and mixing of the MCMC chain were assessed using Tracer version 1.7.2 [27], and effective sample size values greater than 200 were considered sufficient. Summary statistics and ESS values for the BEAST2 analyses are provided in Supplementary Table S3. After excluding the initial 10% of samples as burn-in, a maximum clade credibility tree was generated using TreeAnnotator version 2.7.6. The resulting time-calibrated phylogeny was visualized using FigTree version 1.4, and the estimated node ages were summarized with 95% highest posterior density intervals.

### 2.7. Bayesian Skyline Plot Analysis and Evolutionary Rate Estimation

Bayesian skyline plot analyses were conducted to assess temporal changes in relative genetic diversity within the complete genotype G *HN* gene dataset and within each major phylogenetic group or subgroup. The analyses were performed using BEAST2 version 2.7.6 [24] with the year-stratified deduplicated dataset. The aligned full-length *HN* coding sequences, excluding the terminal stop codon, were used as input. Separate analyses were initially performed for the complete dataset, the Diverse group, Clade 1, Clade 2, Clade2-1, Clade2-2, and Clade2-3. However, the Bayesian skyline analysis of the Diverse group showed unstable posterior behavior in repeated independent BEAST2 runs, as described below, and was therefore not used for biological interpretation. For the Diverse group, repeated independent MCMC runs showed rare extreme excursions in Tree.height, Tree.treeLength, and Bayesian skyline population-size parameters. Therefore, the Diverse-group Bayesian skyline estimates were excluded from biological interpretation and from the main skyline figure.

For each dataset, the nucleotide substitution model was selected using jModelTest2 version 2.1.10 (v20160303) [25]. The Bayesian skyline model was specified as the coalescent tree prior. The molecular clock model was selected from six candidate models, including strict clock, random local clock, optimized relaxed clock, relaxed clock exponential, relaxed clock lognormal, and fast relaxed clock lognormal, using nested sampling as described above [26]. For each dataset, nested-sampling model comparison was used to estimate log marginal likelihoods for the candidate clock models under the Bayesian skyline coalescent prior. The clock model with the highest log marginal likelihood was selected for the final Bayesian skyline and evolutionary-rate analyses. The selected model configurations are summarized in Supplementary Table S2, and the corresponding log marginal likelihood estimates are provided in Supplementary Table S10.

For all datasets, Markov chain Monte Carlo analyses were run for 300 million steps, with parameters sampled every 1000 steps. Convergence and mixing were evaluated using Tracer version 1.7.2 [27], and effective sample size values greater than 200 were considered sufficient. Bayesian skyline plots were summarized and visualized using Tracer, with uncertainty represented by 95% highest posterior density intervals. Molecular evolutionary rates were estimated from the posterior distributions of the mean clock rate parameter obtained in the BEAST2 analyses under the Bayesian skyline coalescent prior. Rate estimates were summarized as posterior means with 95% highest posterior density intervals. Detailed summary statistics and ESS values are provided in Supplementary Table S3.

To assess the potential influence of uneven sampling on the Bayesian skyline analyses, additional sensitivity analyses were performed using downsampled datasets. These analyses focused on the complete genotype G *HN* dataset and the Clade 2 dataset, because the original dataset contained dense sampling of closely related sequences from the 2010s, particularly the heavily sampled 2016–2017 period. For each dataset, two downsampled subsets were prepared: a genetic-distance-based subset, designed to reduce redundancy among closely related sequences, and a temporally adjusted genetic-distance-based subset, designed to further reduce overrepresentation from heavily sampled years while retaining temporal coverage. The complete genotype G dataset was reduced from 346 sequences to 151 and 128 sequences, respectively, and the Clade 2 dataset was reduced from 241 sequences to 80 and 57 sequences, respectively. Sequence inclusion in each downsampled dataset is listed in Supplementary Table S11. Bayesian skyline analyses for the downsampled datasets were performed using the same BEAST2 workflow described above, and the corresponding substitution models, clock models, tree priors, MCMC chain lengths, and logging intervals are summarized in Supplementary Table S12. The results of these sensitivity analyses are shown in Supplementary Figure S1.

### 2.8. Codon-Based Analysis of Selective Pressures in the HN Gene

Codon-based selective pressure analyses were performed using the Datamonkey web server (https://www.datamonkey.org/) (accessed on 24 April 2025) [28]. The unique sequence dataset was used for these analyses to avoid overrepresentation of identical *HN* gene sequences. The aligned full-length *HN* coding sequences, excluding the terminal stop codon, were analyzed for the complete genotype G *HN* dataset and separately for the major phylogenetic groups, including the Diverse group, Clade 1, and Clade 2.

To identify codon sites potentially under positive (diversifying) selection, five approaches were applied: single-likelihood ancestor counting (SLAC) [29], fixed effects likelihood (FEL) applied to all branches [29], FEL applied to internal branches [29], fast unconstrained Bayesian approximation (FUBAR) [30], and mixed effects model of evolution (MEME) [31]. Purifying (negative) selection was assessed using four applicable approaches: SLAC, FEL applied to all branches, FEL applied to internal branches, and FUBAR. MEME was used only for the detection of episodic diversifying selection and was not included in the assessment of purifying selection.

For SLAC, FEL, and MEME, statistical significance was defined as *p* < 0.05. For FUBAR, evidence for selection was inferred when the posterior probability of positive or purifying selection was greater than 0.9. For each codon site, the number of methods supporting positive or purifying selection was counted. The distribution of codon sites supported as being under purifying selection was visualized across the HN protein sequence and mapped onto the representative genotype G HN structural model.

### 2.9. Three-Dimensional Modeling of the MuV HN Protein

Three-dimensional structural models of the MuV HN protein were predicted for representative strains belonging to genotype G lineages. The strains used for structural modeling are listed in Supplementary Table S4.

Structural prediction was performed using LocalColabFold version 1.5.3 installed on a local computer [32]. The workflow was divided into two steps: multiple sequence alignment/template search and structure prediction. First, a3m-format multiple sequence alignment files were generated using colabfold_search with UniRef30 (2302) as the sequence database, PDB100 (230517) as the template database, and colabfold_envdb (202108) as the environmental sequence database. Template search and environmental sequence search were enabled, and the search was performed using MMseqs2 (version 18.8cc5c) with four CPU threads. Second, structure prediction was performed using colabfold_batch with the resulting a3m files and PDB100 template-hit files. The principal options were --amber, --templates, --num-recycle 30, --use-gpu-relax, --pdb-hit-file, and --local-pdb-path. For each *HN* sequence, five candidate models were generated. The representative model was selected by considering model confidence and structural consistency, including predicted local distance difference test (pLDDT), predicted template modeling score (pTM), and root mean square deviation (RMSD). The selected HN structural models were visualized using UCSF ChimeraX version 1.7.1 [33].

### 2.10. Structure-Guided Prediction of Conformational B-Cell Epitopes

Structure-guided conformational B-cell epitope prediction was performed using the genotype G HN protein structural models described above. Four prediction methods were applied: DiscoTope 3.0 using a high-confidence cutoff score of 1.50, corresponding to a recall of up to 30% [34]; ElliPro with a cutoff value of 0.5 [35]; SEPPA 3.0 with a cutoff value of 0.089 [36]; and SEMA with a cutoff value of 0.76 [37]. The URLs and access dates for the web-based prediction tools are provided in Supplementary Table S9.

For each amino acid residue, the number of methods predicting the residue as part of a conformational B-cell epitope was counted. Residues supported by three or more of the four prediction methods were regarded as high-confidence predicted conformational B-cell epitope sites. The number of supporting methods at each residue was visualized as a heatmap using Orange Data Mining version 3.35 [21]. Predicted epitope sites and the number of supporting methods were also mapped onto representative HN structural models and color-coded using UCSF ChimeraX version 1.7.1 [33]. Predicted epitope signals in the N-terminal stalk region were interpreted cautiously because this region showed lower structural confidence in the predicted models and may be overrepresented by structure-based epitope prediction algorithms due to apparent solvent exposure.

## 3. Results

### 3.1. Genetic Divergence and Phylogenetic Separation Among HN Gene Lineages

**Figure 1:**
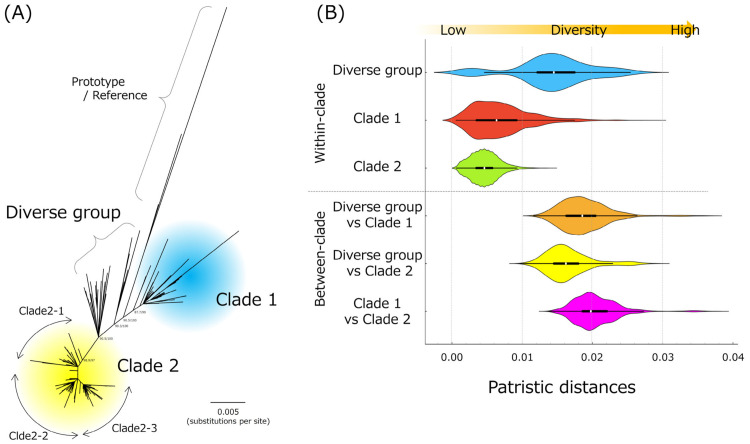
Maximum-likelihood phylogeny and genetic divergence of the *HN* gene lineages in MuV genotype G. (**A**) Maximum-likelihood tree inferred from *HN* gene sequences, showing the phylogenetic separation of Clade 1, the operationally defined Diverse group, and Clade 2. SH-aLRT/ultrafast bootstrap support values are shown for the major nodes used to define the principal genetic groups. Subgroups within Clade 2 are indicated as Clade2-1, Clade2-2, and Clade2-3. Branch lengths are scaled to the number of substitutions per site. (**B**) Patristic distances were calculated to compare genetic divergence within and between the major *HN* gene lineages. Violin plots summarize the distributions of within-lineage distances for the Diverse group, Clade 1, and Clade 2, and between-lineage distances for comparisons among these lineages.

To define the major phylogenetic groups in the MuV *HN* gene dataset, maximum-likelihood phylogeny and phylogenetic distance distributions were examined (Figure 1). The earliest available genotype G strain, detected in 1986, was positioned on an isolated branch separated from the other genotype G sequences. Therefore, this strain was treated as a prototype sequence in subsequent comparisons.

The remaining genotype G sequences were categorized into Clade 1, the Diverse group, and Clade 2 based on the maximum-likelihood tree topology, node support values, patristic distance distributions, and haplotype-network structure. The major node separating Clade 1 from the remaining genotype G *HN* sequences showed high support values of 87.7/99 for SH-aLRT/ultrafast bootstrap, and the node separating Clade 2 from the intervening heterogeneous sequences was also well supported, with values of 98.9/97.

Clade 1 and Clade 2 showed relatively narrow within-group patristic distance distributions, whereas the Diverse group occupied an intermediate position between these groups and exhibited greater within-group genetic variability. These findings indicate that the Diverse group comprises phylogenetically heterogeneous sequences rather than a compact, closely related lineage. Therefore, the Diverse group was not interpreted as a single well-supported monophyletic lineage, but was treated as an operational comparative group comprising sequences located outside Clade 1 and Clade 2. Accordingly, subsequent analyses involving the Diverse group were interpreted cautiously and as descriptive comparisons based on this operational grouping.

### 3.2. Composition and Temporal Distribution of the MuV HN Gene Dataset

**Figure 2:**
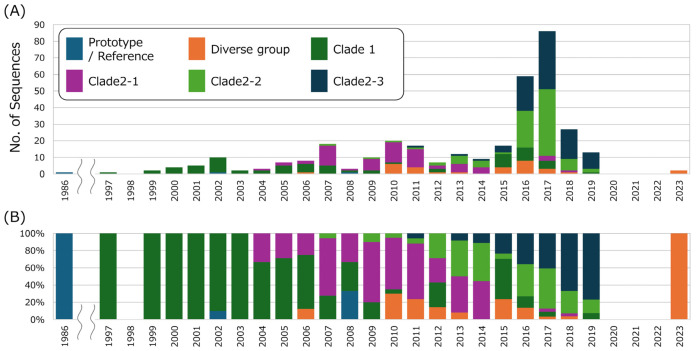
Temporal distribution of MuV genotype G *HN* gene sequences analyzed in this study according to phylogenetic clade. (**A**) Annual number of *HN* gene sequences included in the analysis, stratified by phylogenetic clade. (**B**) Relative annual proportions of sequences assigned to each clade. Colors denote the prototype/reference strains, Diverse group, Clade 1, and the three subgroups within Clade 2. The x-axis indicates the reported year of detection, isolation, or sample collection.

The annual distribution and clade composition of the *HN* gene sequences analyzed in this study are shown in Figure 2. The dataset included one early genotype G strain detected in 1986, followed by sequences reported mainly from 1997 onward. Clade 1 was represented from the late 1990s and continued to be detected intermittently through to 2019. In contrast, Clade 2 was first represented in the dataset in 2004 and subsequently accounted for a large proportion of the available sequences.

Within Clade 2, the relative contribution of each subgroup changed over time. Clade2-1 was mainly observed in the earlier period, whereas Clade2-2 and Clade2-3 became more frequently represented in later years. The number of available sequences increased markedly in 2016 and 2017, largely reflecting sequences associated with outbreaks in the United States [9]. The Diverse group was represented sporadically in the dataset between 2006 and 2023, with sequences absent in several intervening years (Figure 2; Supplementary Table S1).

Because this analysis was based on publicly available full-length *HN* gene sequences, these temporal patterns should be interpreted as the composition of deposited sequences rather than as a direct measure of global circulation or the prevalence of each clade.

### 3.3. Median-Joining Haplotype Network Reveals Diversification of Genotype G HN Gene Lineages

**Figure 3:**
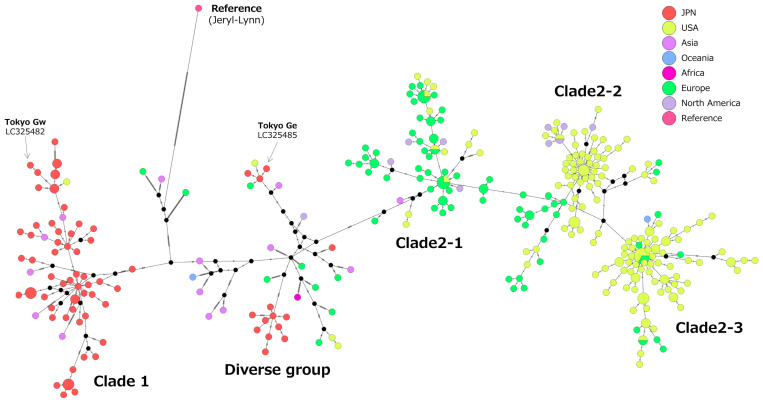
Median-joining haplotype network of MuV *HN* gene sequences. The network was constructed using *HN* gene sequences analyzed in this study to visualize genetic relationships among the identified lineages. Each circle represents a haplotype, and circle size is proportional to the number of sequences sharing the same haplotype. Colors indicate the geographic origin of sequences. Black nodes indicate inferred intermediate haplotypes not observed in the dataset. The genotype A reference strain Jeryl-Lynn (GenBank accession No. AF338106) and representative Japanese genotype G strains, including Tokyo Gw and Tokyo Ge (GenBank accession Nos. LC325482 and LC325485, respectively), are indicated. The major genotype G lineages are labeled as Clade 1, Diverse group, Clade2-1, Clade2-2, and Clade2-3.

A median-joining haplotype network was constructed to visualize genetic relationships among genotype G *HN* gene sequences (Figure 3). The genotype A vaccine strain Jeryl-Lynn was included as a reference for comparison and was connected to the genotype G network through the side corresponding to the Diverse group. The network separated the genotype G sequences into Clade 1, the Diverse group, and Clade 2, consistent with the maximum-likelihood tree.

The Diverse group contained sequences from multiple geographic regions and showed complex haplotype connections with several inferred intermediate nodes, indicating genetic heterogeneity rather than a compact haplotype cluster. This network pattern further supported the operational treatment of the Diverse group as a heterogeneous comparative group located outside Clade 1 and Clade 2, rather than as a single compact monophyletic lineage. Clade 1 was composed mainly of Japanese strains and showed a relatively homogeneous network structure. The representative Tokyo Ge strain was located within the Diverse group, whereas Tokyo Gw was assigned to Clade 1 [38]. Within Clade 2, the three subgroups showed distinct network patterns: Clade2-1 formed a radial structure mainly involving European strains, Clade2-2 formed a dense cluster largely composed of North American strains, and Clade2-3 showed a prominent star-like structure. These patterns indicate that genotype G *HN* gene lineages have diversified into distinct haplotype groups with different geographic and genetic structures within the analyzed dataset.

### 3.4. Clock-like Temporal Structure of the HN Gene Dataset

**Figure 4:**
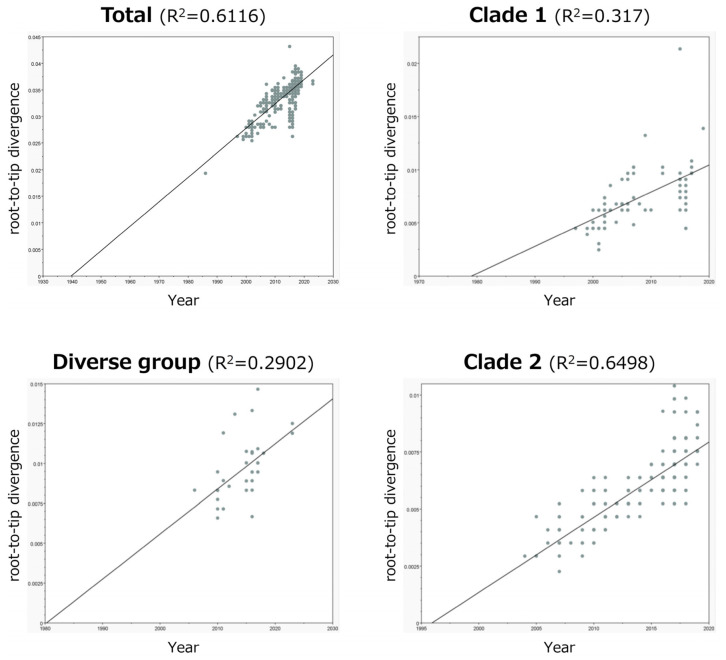
Root-to-tip regression analysis of temporal signal in MuV genotype G *HN* gene datasets. Root-to-tip regression was performed to assess temporal signal prior to Bayesian molecular dating. The complete genotype G *HN* gene dataset and the major phylogenetic groups—Diverse group, Clade 1, and Clade 2—were analyzed separately. Each point represents an individual sequence, with the sampling year plotted on the x-axis and root-to-tip genetic divergence plotted on the y-axis. Regression lines and R^2^ values are shown in each panel. Detailed regression statistics are provided in Supplementary Table S5.

To evaluate the temporal structure of the *HN* gene dataset, root-to-tip regression analysis was performed using TempEst (Figure 4; Supplementary Table S5). In the complete dataset, root-to-tip genetic divergence showed a positive association with sampling year, with an R^2^ value of 0.6116. A similar but slightly stronger temporal pattern was observed for Clade 2, which showed the highest R^2^ value among the analyzed groups (R^2^ = 0.6498). These results indicate that the complete dataset and Clade 2 showed clear temporal structure compatible with subsequent Bayesian molecular dating.

In contrast, the Diverse group and Clade 1 showed weaker temporal signals, with R^2^ values of 0.2902 and 0.3170, respectively. These lower values suggest that molecular dating estimates for these groups should be interpreted with caution. The stronger temporal signal observed in Clade 2 may reflect recent diversification among closely related and relatively densely sampled sequences within the available dataset, consistent with the haplotype network structure described above. Overall, the root-to-tip regression analysis supported the use of time-calibrated phylogenetic analysis while indicating lineage-dependent differences in temporal signal strength.

### 3.5. Time-Calibrated Evolutionary History of Genotype G MuV HN Gene Lineages

**Figure 5:**
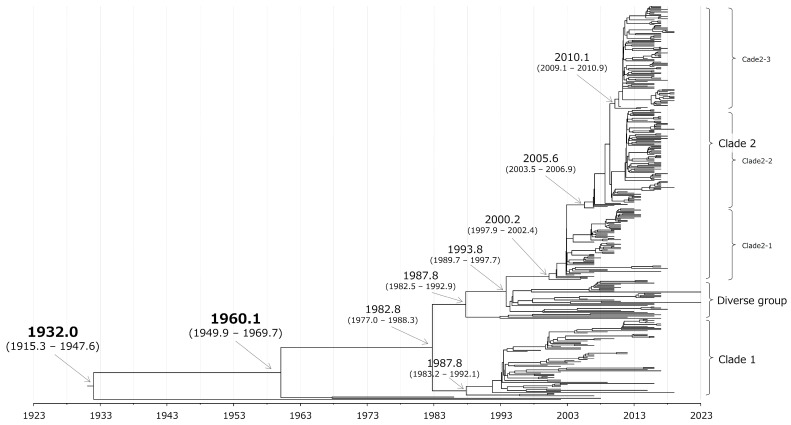
Bayesian time-calibrated phylogeny of MuV genotype G *HN* gene lineages. Bayesian molecular dating was applied to the *HN* gene dataset to reconstruct the temporal diversification of MuV genotype G lineages. The timescale is shown along the x-axis, and branch lengths correspond to elapsed time. Major phylogenetic groups are labeled as Clade 1, Diverse group, and Clade 2, with Clade 2 further subdivided into Clade2-1, Clade2-2, and Clade2-3. Estimated node ages for selected ancestral nodes are indicated together with their 95% highest posterior density intervals.

To infer the evolutionary history of genotype G MuV *HN* gene lineages, a Bayesian time-calibrated phylogeny was reconstructed based on the *HN* gene dataset (Figure 5). Although the earliest available genotype G sequence in this study was detected in 1986, the time to the most recent common ancestor of the sampled genotype G *HN* gene sequences was estimated to be 1932.0 (95% highest posterior density [HPD], 1915.3–1947.6).

The lineage leading to Clade 1 was estimated to have diverged from the other genotype G lineages around 1982.8 (95% HPD, 1977.0–1988.3), and the most recent common ancestor of Clade 1 was estimated to date to 1987.8 (95% HPD, 1983.2–1992.1). The lineage leading to Clade 2 was estimated to have diverged later, around 1993.8 (95% HPD, 1989.7–1997.7), and the most recent common ancestor of Clade 2 was estimated to have existed around 2000.2 (95% HPD, 1997.9–2002.4).

Within Clade 2, the sampled subgroups showed progressively more recent common ancestry during the 2000s. The tMRCA of Clade2-2 was estimated to be 2005.6 (95% HPD, 2003.5–2006.9), whereas that of Clade2-3 was estimated to be 2010.1 (95% HPD, 2009.1–2010.9).

### 3.6. Changes in Relative Genetic Diversity and Lineage-Specific Evolutionary Rates

**Figure 6:**
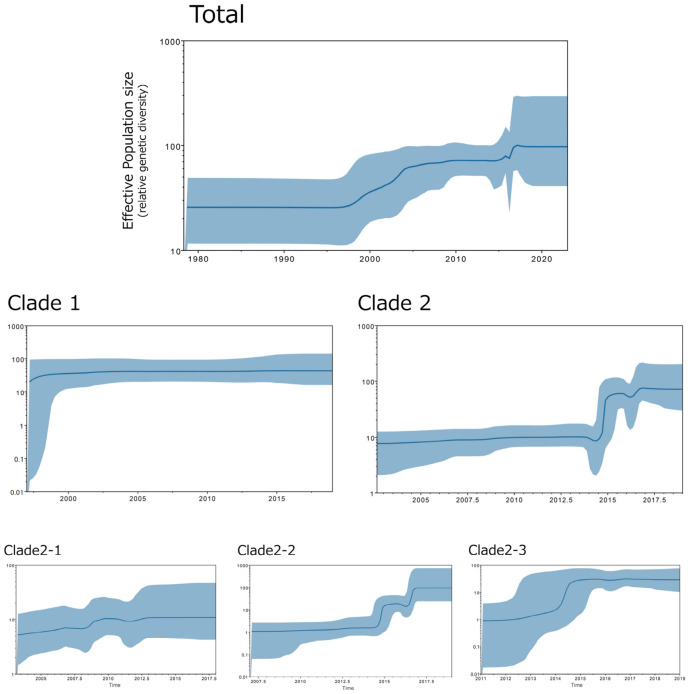
Temporal changes in relative genetic diversity inferred from MuV genotype G *HN* gene sequences. Bayesian skyline plots were used to reconstruct changes in relative genetic diversity over time for the complete *HN* gene dataset and for selected genetic groups or subgroups. Separate plots are shown for the complete dataset, Clade 1, Clade 2, Clade2-1, Clade2-2, and Clade2-3. Bayesian skyline estimates for the Diverse group were excluded from this figure and from biological interpretation because repeated independent BEAST2 analyses showed unstable posterior behavior, as described in Section 2.7. The central line indicates the median estimate, while the shaded area represents the 95% highest posterior density interval. The x-axis denotes calendar year, and the y-axis shows inferred relative genetic diversity on a logarithmic scale.

Bayesian skyline plot analyses were performed to examine temporal changes in relative genetic diversity in the complete genotype G *HN* gene dataset and in each major genetic group or subgroup (Figure 6). In the complete dataset, relative genetic diversity increased around the late 1990s to early 2000s and then remained at a relatively high level. However, this complete-dataset pattern was interpreted cautiously because the dataset combined multiple genetic groups and was affected by uneven temporal and geographic sampling. Clade 1 showed no marked temporal increase or decrease, suggesting relatively stable relative genetic diversity within the available sequence dataset. The Bayesian skyline estimates for the Diverse group were not used for biological interpretation because this group was phylogenetically heterogeneous, showed a weaker temporal signal, and exhibited unstable posterior behavior in repeated BEAST2 analyses. In contrast, Clade 2 showed an overall increase in relative genetic diversity, although the magnitude and timing of this increase were potentially affected by sampling structure. At the subgroup level, increases were observed around the periods corresponding to the estimated emergence and diversification of Clade2-2 and Clade2-3.

To evaluate whether these Bayesian skyline patterns were influenced by uneven sampling, sensitivity analyses were performed using genetic-distance-based and temporally adjusted downsampled datasets for the complete genotype G dataset and for Clade 2 (Supplementary Figure S1; Supplementary Tables S11 and S12). In the complete genotype G dataset, the recent increase observed in the original dataset was attenuated after downsampling, indicating that the complete-dataset skyline was influenced by dense sampling of Clade 2 sequences from the 2010s, particularly the heavily sampled 2016–2017 period. In contrast, when Clade 2 was analyzed separately, an overall increase in relative genetic diversity was retained in both downsampled datasets, although the magnitude and timing of the increase varied among datasets. These results suggest that the Clade 2 skyline pattern was not fully explained by sampling bias alone, but should nevertheless be interpreted as a sampling-sensitive increase in relative genetic diversity within the available sequence dataset rather than as direct evidence of population-level viral expansion.

**Figure 7:**
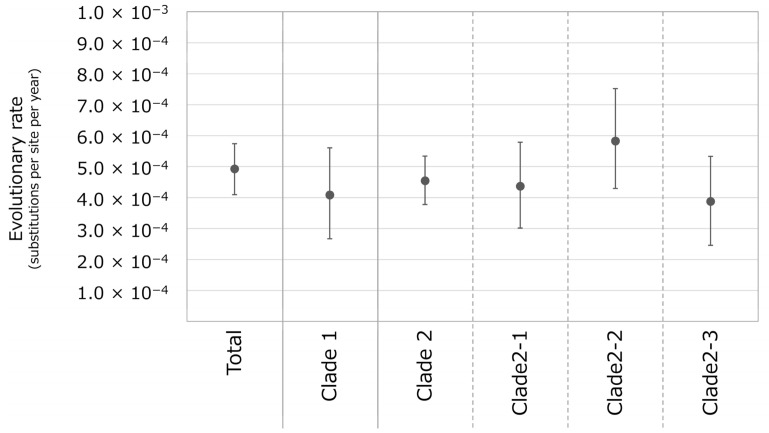
Lineage-specific evolutionary rates of the MuV *HN* gene. Substitution rates were inferred separately for the complete genotype G *HN* gene dataset and for each major lineage or sublineage. Each point represents the posterior mean evolutionary rate, and vertical bars indicate the 95% highest posterior density interval. Rates are expressed as substitutions per site per year.

The mean evolutionary rate of the complete genotype G *HN* gene dataset was estimated to be 4.925 × 10^−4^ substitutions/site/year (95% highest posterior density [HPD], 4.0937 × 10^−4^–5.7404 × 10^−4^) (Figure 7). Among the major groups, the Diverse group showed the lowest mean rate at 2.260 × 10^−4^ substitutions/site/year (95% HPD, 6.5322 × 10^−5^–3.9458 × 10^−4^), whereas Clade 1 and Clade 2 showed comparable rates of 4.083 × 10^−4^ (95% HPD, 2.6685 × 10^−4^–5.6101 × 10^−4^) and 4.543 × 10^−4^ (95% HPD, 3.7773 × 10^−4^–5.3425 × 10^−4^) substitutions/site/year, respectively. Within Clade 2, Clade2-2 showed the highest mean rate (5.827 × 10^−4^ substitutions/site/year), followed by Clade2-1 and Clade2-3. However, the 95% HPD intervals overlapped among lineages, indicating that differences in evolutionary rate should be interpreted cautiously. Together, these results suggest that Clade 2 showed more pronounced, but sampling-sensitive, temporal changes in relative genetic diversity than Clade 1, whereas lineage-specific rate differences were moderate and should be interpreted cautiously, particularly for the operationally defined Diverse group.

### 3.7. Codon-Based Selection Analyses Indicate Predominant Purifying Selection in the HN Gene

Codon-based selection pressure analyses were performed to evaluate whether the *HN* gene was subject to positive or purifying selection (Supplementary Table S6). Detailed site-level results of the selection analyses are provided in Supplementary Table S7.

Positive selection was assessed using five algorithms, including FEL applied to all branches, FEL applied to internal branches, FUBAR, MEME, and SLAC. Although a small number of codon sites were identified as being under positive selection by a single method, no sites were consistently supported by two or more algorithms. Thus, there was no robust evidence of positive selection supported by multiple methods for the *HN* gene.

**Figure 8:**
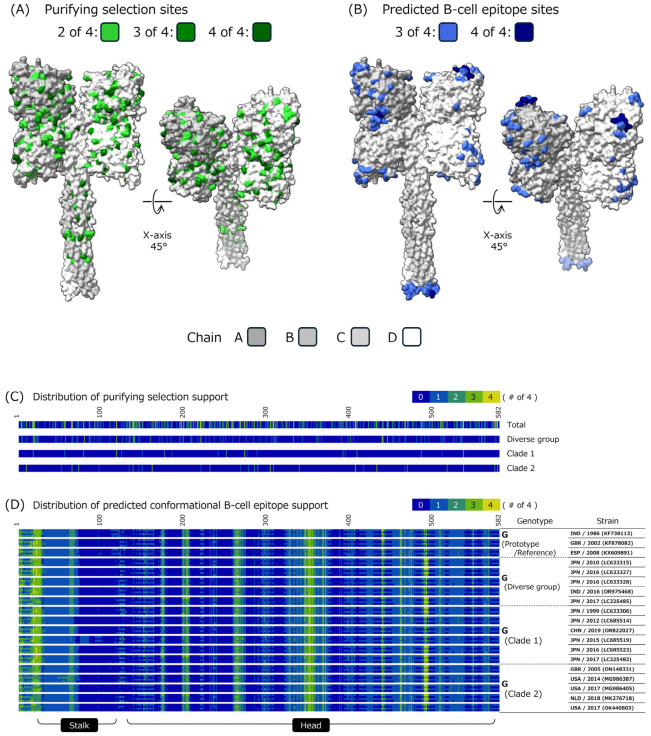
Structure-guided mapping of purifying selection sites and predicted B-cell epitopes in the MuV genotype G HN protein. (**A**) Codon sites supported as being under purifying selection were mapped onto a three-dimensional model of the HN protein from a representative genotype G strain, Tokyo Gw (GenBank accession No. LC325482). Residues are colored according to the number of applicable selection methods supporting purifying selection at each site. (**B**) Predicted conformational B-cell epitopes were mapped onto the same HN structural model. Residues are colored according to the number of prediction methods supporting each residue as a predicted epitope site. (**C**) Distribution of purifying selection support across the HN protein in the complete genotype G dataset and in the major phylogenetic groups (Diverse group, Clade 1, and Clade 2). The color scale indicates the number of applicable selection methods supporting purifying selection at each site. (**D**) Distribution of supported predicted conformational B-cell epitopes across the HN protein among representative MuV strains from genotype G lineages. The color scale indicates the number of prediction methods supporting each residue as a predicted epitope site. The stalk and head regions of the HN protein are indicated below the heatmap.

In contrast, purifying selection was supported at many codon sites when assessed using four applicable algorithms, because MEME detects episodic diversifying selection and was therefore not included in the assessment of purifying selection. Several sites were supported by multiple methods, indicating that a substantial proportion of the *HN* gene is subject to evolutionary constraint. Purifying selection sites supported by multiple methods were mapped onto a three-dimensional HN protein model of a representative genotype G strain (Figure 8A). The distribution of purifying selection support across the HN protein was also visualized as a heatmap (Figure 8C). Sites inferred to be under purifying selection were broadly distributed across the HN protein, without obvious clustering in a particular protein region or a clear clade-specific pattern (Figure 8C; Supplementary Table S7). Overall, these findings indicate that the evolution of the MuV *HN* gene is dominated by purifying selection rather than widespread positive selection.

### 3.8. Structural Mapping of Predicted B-Cell Epitopes in the HN Protein of MuV Genotype G

To examine the distribution of predicted conformational B-cell epitopes in the HN protein of MuV genotype G, conformational B-cell epitope prediction was performed for representative HN proteins from genotype G lineages using four prediction methods based on different theoretical frameworks (Figure 8). Detailed site-level results of the epitope prediction analyses are provided in Supplementary Table S7.

Predicted epitope sites supported by multiple methods were mapped onto a three-dimensional HN protein model of a representative genotype G strain (Figure 8B). Predicted epitope sites supported by three or more methods were observed in the head region of the HN protein.

The distribution of predicted epitope support across the HN protein was further visualized as a heatmap (Figure 8D). The overall pattern of predicted epitope support was broadly similar among the representative strains, with a region around amino acid position 350 showing relatively strong support from multiple methods. These findings suggest that the overall distribution of predicted B-cell epitope regions in the HN protein is largely shared among representative strains.

## 4. Discussion

By applying a range of bioinformatics approaches, we comprehensively characterized the genetic divergence, temporal dynamics, phylodynamic patterns, selection pressures, and predicted epitope features of the MuV genotype G *HN* gene and encoded HN protein. The results indicate that genotype G *HN* gene sequences are not phylogenetically uniform, but can be categorized into Clade 1, the operationally defined Diverse group, and Clade 2, as shown by the maximum-likelihood phylogeny and patristic distance analyses (Figure 1). Clade 2 exhibited relatively shallow internal diversification and subgroup structure, suggesting recent diversification from closely related ancestral viruses. In contrast, the Diverse group represented phylogenetically heterogeneous sequences located outside Clade 1 and Clade 2, rather than a single well-supported monophyletic lineage. Thus, the evolutionary patterns inferred in this study should be interpreted according to the nature of each genetic group and the sampling structure of the available dataset.

First, the phylogenetic distance analysis, maximum-likelihood tree, median-joining network, and Bayesian time-calibrated phylogeny consistently supported the separation of Clade 1, the Diverse group, and Clade 2 (Figure 1, Figure 3 and Figure 5). Clade 1 was represented mainly by Japanese strains and appeared to maintain a relatively structured lineage over an extended period. This pattern may be compatible with long-term endemic circulation or the persistence of multiple related lineages within a geographically structured population. In contrast, Clade 2 showed lower overall within-clade divergence and shallow internal branching while forming distinct subgroups. This suggests that Clade 2 may have expanded more recently from a limited ancestral genetic background, followed by subgroup-level diversification. Previous studies have separately described long-term genotype G circulation in Japan and outbreak-associated genotype G lineages in the United States [9,15,38]. Building on these region-specific studies, the present analysis placed globally available genotype G *HN* sequences into a unified comparative framework and highlighted contrasting evolutionary signatures among regionally enriched lineages.

The temporal distribution of the dataset further demonstrated changes in the composition of genotype G *HN* lineages over time (Figure 2). Clade 1 strains were detected from the late 1990s onward and appeared intermittently over time, whereas Clade 2 strains became increasingly represented in the dataset after the 2000s. In particular, the increased number of available sequences in 2016 and 2017 largely reflected outbreak-associated sequences deposited from the United States [9,15]. However, because the present analysis was based on publicly available full-length *HN* gene sequences, these temporal patterns should be interpreted as the composition of deposited sequences rather than as direct evidence of global circulation or clade prevalence [39].

Next, the median-joining haplotype network provided additional support for the distinct genetic architectures of genotype G lineages (Figure 3). The Diverse group showed complex haplotype connections with several inferred intermediate nodes, suggesting genetic heterogeneity rather than a compact lineage structure. Clade 1 showed a relatively homogeneous network structure, mainly involving Japanese strains, whereas Clade 2 displayed subgroup-specific network patterns. In particular, the radial or star-like configurations observed in some Clade 2 subgroups are compatible with relatively recent expansion from closely related ancestral haplotypes [22,40]. These network patterns reinforce the interpretation that genotype G lineages, as defined by *HN* gene variation, have diversified through distinct evolutionary and epidemiological processes.

The relatively homogeneous and structured pattern of Clade 1 should also be interpreted in the context of Japan’s unique mumps vaccination history and surveillance structure. In Japan, routine MMR vaccination was discontinued in 1993 because of concerns regarding vaccine-associated aseptic meningitis, and monovalent mumps vaccination has subsequently been used on a voluntary basis [41,42,43]. This public health background may have allowed MuV lineages to circulate locally over extended periods under vaccination coverage and transmission conditions that differed from those in countries with routine two-dose MMR programs. Therefore, the Clade 1 pattern observed in this study may reflect local endemic circulation, regional vaccination policy, uneven geographic sampling, and intensive local sequencing efforts, rather than intrinsic viral evolutionary properties alone. Accordingly, the phylodynamic features of Clade 1 should be interpreted cautiously as patterns observed in the available sequence dataset.

These network-based findings also highlight the potential utility of *HN* gene sequence analysis as a complementary approach for MuV molecular epidemiology. *SH* gene sequencing remains the standard approach for MuV genotyping and provides a practical framework for routine molecular surveillance [44,45]. However, in settings where most circulating strains belong to the same genotype, such as genotype G, genotype assignment alone may provide limited resolution for evaluating relationships among closely related strains. In the present analysis, full-length *HN* gene sequences resolved genotype G viruses into distinct haplotype groups and subgroup-specific network patterns, suggesting that *HN* gene data can provide additional resolution for within-genotype molecular epidemiology. Therefore, the accumulation of *HN* gene sequence data, together with *SH* gene-based genotype information, may improve molecular surveillance of MuV genotype G and support more detailed local public-health investigations of transmission and lineage diversification.

Root-to-tip regression analysis demonstrated that the complete genotype G *HN* gene dataset and Clade 2 had a clear temporal structure, supporting the application of Bayesian molecular dating (Figure 4) [23,46]. In contrast, the Diverse group and Clade 1 showed weaker temporal signals, suggesting that molecular dating estimates for these lineages should be interpreted with caution [47]. The stronger temporal signal observed in Clade 2 likely reflects its more recent diversification and denser sampling within the available dataset. Therefore, the temporal estimates for Clade 2 may be more robust than those for lineages with weaker clock-like structures.

The Bayesian time-calibrated phylogeny suggested that the most recent common ancestor of the analyzed genotype G *HN* gene sequences dated back to approximately 1932, whereas the major lineages appeared to have diversified mainly from the late twentieth century onward (Figure 5). This finding indicates that the ancestral genotype G *HN* lineage may have existed long before the first available genotype G sequence analyzed in this study. However, the long interval between the inferred ancestral node and the earliest sampled genotype G strain likely reflects the limited availability of historical sequence data rather than continuous documentation of genotype G circulation [48,49,50]. Nevertheless, this temporal framework is informative because it places the more recent diversification of Clade 1 and Clade 2 within a deeper, previously unsampled evolutionary background of genotype G *HN* lineages.

The estimated divergence times also suggested different evolutionary histories among the major lineages. The lineage leading to Clade 1 was estimated to have diverged earlier than the lineage leading to Clade 2, whereas the most recent common ancestor of Clade 2 was estimated to date to approximately 2000 (Figure 5). Within Clade 2, Clade2-2 and Clade2-3 showed more recent common ancestry during the 2000s. These findings are consistent with the phylogenetic and network-based observations that Clade 2 represents a relatively recently expanded lineage with shallow internal diversification.

Bayesian skyline plot analysis suggested lineage-dependent differences in relative genetic diversity, but these phylodynamic patterns should be interpreted cautiously because the analyses were based on publicly available sequences with uneven temporal and geographic sampling (Figure 6) [51,52]. In the complete genotype G *HN* dataset, relative genetic diversity increased around the late 1990s to early 2000s and then remained at a relatively high level. However, this complete-dataset pattern should not be interpreted as direct evidence of global genotype G population expansion because the dataset combined multiple genetic groups and was influenced by dense sampling of Clade 2 sequences from the 2010s. In addition, Bayesian skyline estimates for the Diverse group were not used for biological interpretation because this operational group was phylogenetically heterogeneous, showed a weaker temporal signal, and exhibited unstable posterior behavior in repeated BEAST2 analyses.

The downsampling sensitivity analyses further supported this cautious interpretation. In the complete genotype G dataset, the recent increase observed in the original skyline plot was attenuated after genetic-distance-based and temporally adjusted downsampling, indicating that the complete-dataset skyline was partly influenced by sampling structure. In contrast, when Clade 2 was analyzed separately, an overall increase in relative genetic diversity was retained after downsampling, although the magnitude and timing of the increase varied among datasets. Thus, the Clade 2 pattern may reflect an increase in relative genetic diversity within the available sequence dataset, but it should be regarded as a sampling-sensitive phylodynamic signal rather than direct evidence of population-level viral expansion. Regional differences in epidemiological background, population immunity, outbreak intensity, and sequencing activity may have contributed to these patterns [38,53,54]. Therefore, these findings should be considered a working hypothesis to guide future studies integrating sequence data with epidemiological, serological, and immunological information, rather than evidence of a direct causal relationship between specific epidemiological factors and lineage diversification [39,55].

Next, the estimated evolutionary rate of the complete genotype G *HN* gene dataset was approximately 4.9 × 10^−4^ substitutions/site/year (Figure 7). Although lineage-specific rates varied, the 95% highest posterior density intervals overlapped among groups, indicating that these differences should be interpreted cautiously [49]. Clade2-2 showed the highest mean rate, but this may reflect recent outbreak-associated sampling, short-term evolutionary dynamics, or stochastic effects rather than a stable biological difference in substitution rate [50,56]. Overall, the data suggest moderate lineage-specific variation in evolutionary rate while supporting broadly comparable substitution-rate ranges among major genotype G lineages. The evolutionary rate estimated for the MuV genotype G *HN* gene was comparable to that reported for the HPIV4 *F* gene, whereas higher evolutionary rates have been reported for norovirus *VP1* genes [57,58]. Although these genes encode exposed structural proteins that can serve as targets of host immunity, this comparison suggests that antigenically relevant protein-coding genes do not necessarily evolve at uniformly high rates. Because viral evolutionary rates are influenced by multiple biological and epidemiological factors, direct comparisons across different viruses and genes should be interpreted cautiously [59]. Nevertheless, the relatively moderate evolutionary rate of the MuV *HN* gene may reflect functional constraints required to maintain receptor-binding and neuraminidase activities.

Codon-based selection analyses indicated that the *HN* gene is predominantly subject to purifying selection (Supplementary Table S6) [28,29]. Given that the HN protein is a major surface antigen and a target of neutralizing antibodies, positive (diversifying) selection at antigenically relevant sites might have been expected. However, although a small number of codon sites were identified as positively selected by individual methods, no sites were consistently supported by multiple algorithms. In contrast, many sites were inferred to be under purifying selection, suggesting that functional constraints play a major role in *HN* gene and protein evolution [60]. This is biologically plausible because the HN protein is involved in multiple functions, such as receptor binding, neuraminidase activity, and activation of the fusion protein during viral entry [4,5,61]. Thus, although genotype G *HN* lineages have diversified phylogenetically, their evolution appears to be constrained by the requirement to maintain essential protein functions.

The predominance of purifying selection in genotype G HN is notable because HN is an exposed surface glycoprotein and a major target of neutralizing antibodies. This apparently paradoxical pattern may be explained by several non-mutually exclusive mechanisms. First, HN is not only an antigenic protein but also a multifunctional glycoprotein involved in receptor binding, neuraminidase activity, and the viral entry process. Therefore, amino acid substitutions, even in surface-exposed regions, may be tolerated only within a limited structural and functional range. Second, immune pressure against MuV HN may be heterogeneous among populations, incomplete, or insufficiently directional to drive consistent selective sweeps detectable across the globally available genotype G *HN* dataset. Third, the broadly similar distribution of predicted conformational B-cell epitope regions should not be interpreted as evidence of antigenic equivalence, but may instead reflect the structural constraints imposed on exposed regions of the HN protein. Together, these considerations suggest that the apparent conservation of genotype G HN may reflect a balance between immune exposure and strong structural-functional constraints. However, this hypothesis cannot be tested directly using sequence-based and in silico structural analyses alone, and experimental antigenic and functional studies will be required to clarify the underlying mechanisms.

In addition to these protein-level constraints, codon conservation in negative-strand RNA viruses may also reflect RNA-level constraints associated with viral RNA synthesis. In negative-strand RNA viruses, genome and antigenome synthesis occurs in the context of ribonucleoprotein complexes, and codon usage or synonymous nucleotide changes may influence RNA synthesis efficiency or related sequence-context constraints. Therefore, the predominance of purifying selection observed in the *HN* gene should not be attributed solely to the maintenance of HN protein function, but may also reflect constraints acting at the level of viral RNA synthesis and genome organization [62]. Future studies that explicitly examine synonymous-site evolution, codon usage, RNA sequence context, and viral replication phenotypes will be needed to clarify the contribution of RNA-level constraints to *HN* gene conservation.

The epitope prediction analysis further helped relate predicted epitope regions to the distribution of purifying selection sites in the HN protein. Predicted conformational B-cell epitope support was mainly observed in the HN head region and showed broadly similar distribution patterns among representative genotype G strains (Figure 8). In contrast, purifying selection support was distributed across the HN protein rather than being concentrated in a particular subregion. Taken together, these findings suggest that the genotype G HN protein contains predicted epitope regions while remaining subject to broad functional constraints. This pattern may indicate that, within genotype G, HN protein evolution has proceeded without clear evidence of widespread positive (diversifying) selection, including in predicted epitope regions. Predicted conformational B-cell epitope support was mainly observed in the HN head region and showed broadly similar distribution patterns among representative genotype G strains. However, these results should be interpreted cautiously. The present analysis used structure-guided conformational B-cell epitope prediction tools rather than purely linear epitope-prediction methods; nevertheless, in silico epitope prediction cannot fully capture the complexity of antibody recognition of native HN proteins. Therefore, the broadly similar predicted epitope patterns should not be interpreted as evidence of antigenic equivalence, equivalent neutralization sensitivity, absence of immune escape, or stable vaccine-antigen targets among genotype G strains [63]. These predicted epitope-containing regions should instead be regarded as candidate regions for future experimental antigenic and immunological validation. Functional studies, including serum neutralization assays, antibody-binding experiments, and experimental validation using recombinant HN proteins or infectious clones, will be required to clarify the antigenic and immunological implications of these findings [11,64]. Nevertheless, the present findings may help guide future experimental studies of the antigenic and immunological properties of genotype G HN proteins.

This study has several limitations. First, the dataset consisted of publicly available full-length *HN* gene sequences, and therefore the temporal and geographic distributions reflect sequence deposition patterns rather than unbiased global circulation [39,55]. Second, within the dataset analyzed in this study, sampling was uneven across years and regions, which may affect estimates of genetic diversity, lineage expansion, and evolutionary rate [65,66]. Third, some lineages, particularly the Diverse group and Clade 1, showed weaker temporal signals, and their molecular dating estimates should be interpreted cautiously [23,47]. Fourth, the analysis focused on the *HN* gene alone; integration with whole-genome data, including the *F* gene and other genomic regions, would provide a more comprehensive understanding of MuV evolution. Finally, the structural epitope analysis was predictive and requires experimental validation [63].

In conclusion, the genotype G MuV *HN* gene shows phylogenetic heterogeneity and can be categorized into Clade 1, the operationally defined Diverse group, and Clade 2, with group-specific evolutionary patterns in the available sequence dataset. Clade 1 may represent a relatively stable and geographically structured genetic group, whereas Clade 2 showed more recent diversification and a sampling-sensitive overall increase in relative genetic diversity. Despite this lineage differentiation, the *HN* gene is largely conserved and appears to be shaped predominantly by purifying selection. These findings provide a framework for understanding the molecular evolution of genotype G MuV *HN* sequences and highlight the need for integrated genomic, epidemiological, serological, and vaccination data to clarify the drivers of MuV lineage diversification. This perspective is consistent with the broader concept of integrative viral bioinformatics, which links viral sequence variation with structural, epidemiological, and public-health-relevant interpretation [67].

## Data Availability

All sequence data analyzed in this study are publicly available, and accession numbers are listed in Supplementary Table S1.

## Supplementary Materials

The following supporting information can be downloaded at: https://www.mdpi.com/article/10.3390/microorganisms14071597/s1, Table S1: List of Mumps virus genotype G strains retrieved from NCBI GenBank used in this study; Table S2: Parameters used in the Bayesian Markov chain Monte Carlo (MCMC) analyses; Table S3: Summary statistics and effective sample size (ESS) values for each parameter from the BEAST analyses; Table S4: Representative mumps virus genotype G strains used for HN protein structural modeling and epitope prediction analyses; Table S5: Summary statistics of root-to-tip regression analyses used to assess temporal signal in MuV genotype G *HN* gene datasets; Table S6: Distribution of *HN* gene codon sites according to the number of selection methods supporting positive selection or purifying selection; Table S7: Positive and purifying selection sites and predicted conformational B-cell epitopes in the HN protein; Table S8: Sequence retrieval, filtering, and dataset construction workflow for MuV genotype G *HN* gene analysis; Table S9: The URLs and access dates for the web-based prediction tools; Table S10: Nested-sampling comparison of molecular clock models and coalescent tree priors used for BEAST2 analyses; Table S11: Sequence inclusion in the genetic-distance-based and temporally adjusted downsampled datasets used for Bayesian skyline sensitivity analyses; Table S12: BEAST2 model configurations and MCMC settings for Bayesian skyline analyses of the downsampled datasets; Figure S1: Sensitivity analysis of Bayesian skyline plots using genetic-distance-based and temporally adjusted downsampled datasets.
