# Supplementary material for: Lineage Diversification and Evolutionary Dynamics of the Hemagglutinin–Neuraminidase Gene in Mumps Virus Genotype G
Source: Microorganisms. 2026 Jul 22;14(7):1597. doi: 10.3390/microorganisms14071597 (PMC13414252; doi:10.3390/microorganisms14071597)
Supplement: Supplementary file 1 [file microorganisms-14-01597-s001.zip › MuV_GenotypeG_HN_SupplementaryFigures.pdf]

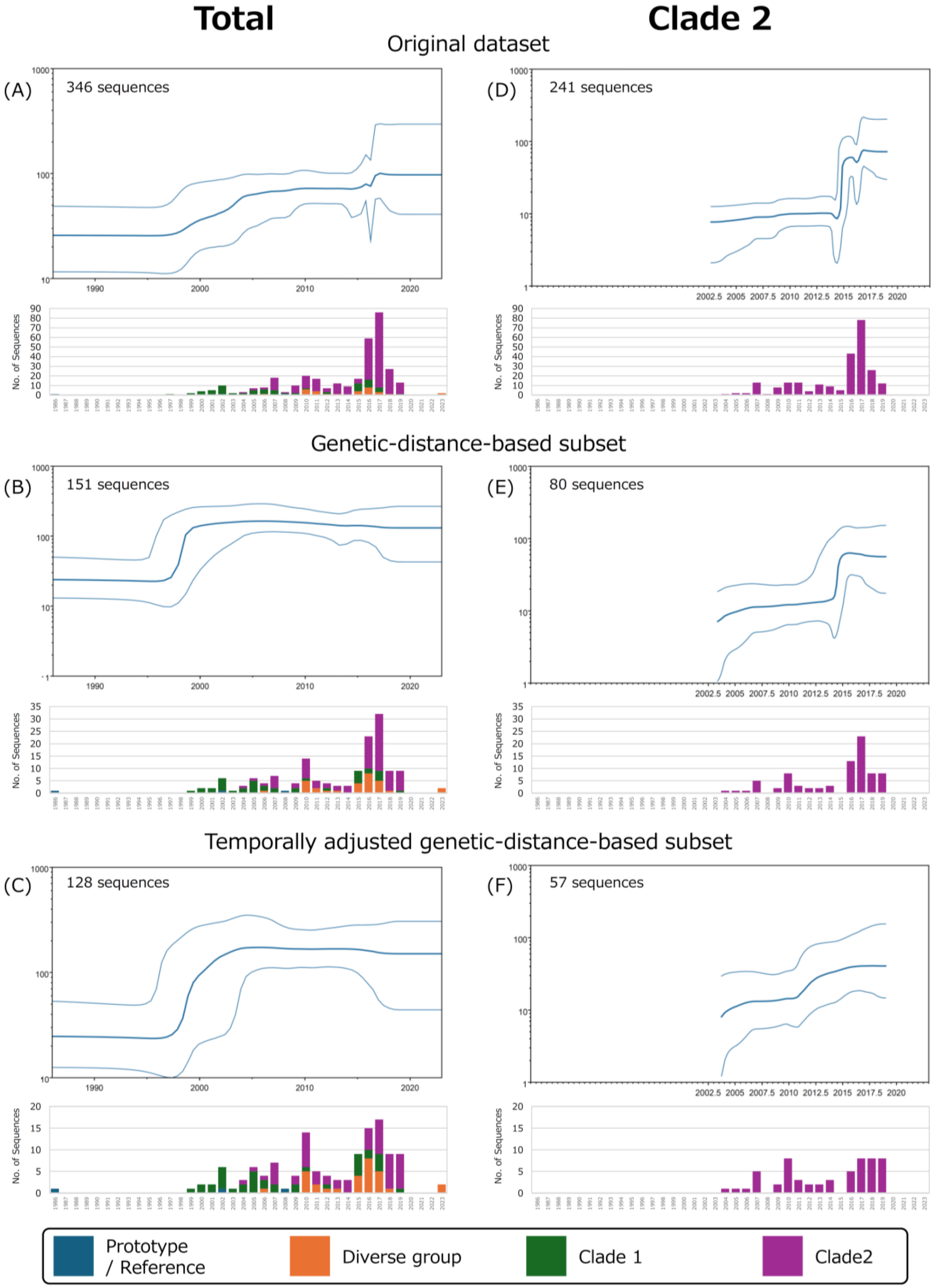
Total
Clade 2
Original dataset
(A)
346 sequences
(D)
241 sequences
Genetic-distance-based subset
(B)
151 sequences
(E)
80 sequences
Temporally adjusted genetic-distance-based subset
(C)
128 sequences
(F)
57 sequences
No. of Sequences
Prototype / Reference
Diverse group
Clade 1
Clade2

Supplementary Figure S1

Sensitivity analysis of Bayesian skyline plots using genetic-distance-based and temporally adjusted downsampled datasets.

Bayesian skyline plots were compared among the original datasets, genetic-distance-based subsets, and temporally adjusted genetic-distance-based subsets to evaluate the influence of uneven sampling on inferred changes in relative genetic diversity. Analyses were performed for the complete genotype G HN dataset (A–C) and the Clade 2 dataset (D–F). The genetic-distance-based subsets were generated to reduce redundancy among closely related sequences, whereas the temporally adjusted genetic-distance-based subsets were further adjusted to reduce overrepresentation from heavily sampled years, particularly the 2016–2017 period. Bar plots below each skyline plot show the temporal distribution and group composition of the sequences included in each analysis. Solid lines indicate posterior median estimates, and shaded areas indicate 95% highest posterior density intervals.
